# The five-item modified frailty index predicts long-term outcomes in elderly patients undergoing colorectal cancer surgery

**DOI:** 10.1186/s12957-023-03150-2

**Published:** 2023-08-26

**Authors:** Toshiro Ogata, Yoshihiko Sadakari, Hiroyuki Nakane, Kazuhiro Koikawa, Hiroki Kanno, Ryo Kohata, Kayoko Endo, Takao Tsukahara, Koichiro Shimonaga, Kazuhisa Kaneshiro, Gentaro Hirokata, Takeshi Aoyagi, Chiyo Tsutsumi, Masahiko Taniguchi

**Affiliations:** 1 Department of Surgery, St. Mary’s Hospital, Tsubukuhonmachi, Kurume, Fukuoka 830-8543 Japan; 2 Department of Medical Biostatistics, St. Mary’s Hospital, 422 Tsubukuhonmachi, Kurume, Fukuoka 830-8543 Japan

**Keywords:** Five-item modified frailty index, Colorectal cancer surgery, Non-colorectal cancer-related death, Colorectal cancer death, Long-term survival

## Abstract

**Background:**

Frailty has been globally recognized as a predictor of adverse postoperative outcomes. Frailty assessment using the five-factor modified frailty index (5-mFI) has recently gained traction; however, long-term outcomes are unknown in colorectal cancer (CRC) surgery. This study aimed to investigate whether the 5-mFI predicted long-term survival and cause of death on the basis of frailty severity in elderly patients who underwent CRC surgery and to determine the risk factors for mortality.

**Methods:**

A total of 299 patients underwent CRC surgery with curative intent between January 2013 and December 2017. Patients were divided into three groups by the 5-mFI score: group 1 (5-mFI: 0 or 1; *n* = 164): no frailty; group 2 (5-mFI: 2; *n* = 91): moderate frailty; and group 3 (5-mFI: ≥ 3; *n* = 44): severe frailty. Clinicopathological variables, namely comorbidities, 5-mFI, prognostic nutrition index, operative/postoperative data, and outcome, including cause of death, were compared between the three groups. To identify factors associated with death from CRC- and non-CRC-related causes, univariate and multivariate analyses using a Cox regression model were performed.

**Results:**

The immediate postoperative morbidity of patients with Clavien–Dindo grade ≥ III complications (9.1%) in group 3 was not significantly different from that in group 1 (9.1%) or group 2 (14.3%); however, the 30-day mortality rate (4.5%) in group 3 was significantly higher. Long-term disease-free survival was similar between frailty groups, suggesting that CRC surgery provides oncological benefit to patients irrespective of frailty. The 5-year survival rates in groups 1, 2, and 3 were 83.5%, 71.2%, and 47.9%, respectively, showing a significantly lower survival rate as frailty advanced. Sixty percent of the deaths in frail patients were due to respiratory failure and cardiovascular diseases. Multivariate analysis demonstrated that advanced age, higher 5-mFI score, and longer postoperative hospital stay were risk factors for mortality unrelated to CRC. Multivariate analysis also revealed that advanced tumor stage, carcinoembryonic antigen ≥ 5 ng/ml, undifferentiated tumor, and R1 resection were risk factors for CRC-related mortality.

**Conclusions:**

The 5-mFI score can predict postoperative short- and long-term outcomes and risk factors for mortality unrelated to CRC. Additionally, long-term survival was negatively associated with the 5-mFI score.

## Background

Frailty is a geriatric syndrome characterized by physiological decline of multiple organ systems and increased vulnerability to stressor events [1]. With growing aging populations worldwide, frailty has recently been recognized as an important risk factor for adverse outcomes in elderly patients after surgery [1, 2]. Frailty is associated with postoperative complications, higher mortality, longer hospital stays, higher readmission rates, loss of independence, and increased healthcare costs [2–5]. Although frailty has become a tool for risk stratification in older adults undergoing surgery [6], a global standard for simple and reliable assessment of frailty has not yet been established.

In 2001, a physical phenotype model by Fried et al. [7] and an accumulating deficits model by Mitnitski et al. [8] were developed. However, both models are cumbersome, complex, and time-consuming because they comprise 5–92 preoperative variables that can impact decision making immediately before surgery [7–9]. Subsequently, the Canadian Study of Health and Aging (CSHA) introduced a less complex frailty index (CSHA-FI) that is composed of 70 clinically practicable items [10]. In 2013, the American College of Surgeons National Surgical Quality Improvement Program provided a modified frailty index by extracting 11 variables from the original CSHA-FI [9, 11]. Similar to the CSHA-FI, the 11-item modified frailty index (11-mFI) was proven to be a valid predictor of postoperative morbidity and mortality [9, 12–14]. Recently, the five-item modified frailty index (5-mFI) using a subset of five variables extracted from the 11-mFI was introduced [15–17]. The 5-mFI comprises four comorbid conditions (pulmonary disease, congestive heart failure, diabetes mellitus, and hypertension) and one functional status [16–18]. Similar to the original 11-mFI, the 5-mFI has been proven to be an acceptable predictor of outcome in various surgeries, namely paraesophageal hernia repair, multiorgan resections, trauma, nephrectomy, breast reconstruction, spine surgery, and complex head and neck surgery [15, 16, 18–23].

Increased population aging has resulted in the need for elderly patients to undergo colorectal cancer (CRC) surgery. Although these patients have higher morbidity and reduced survival [24] compared with younger patients, the influence of frailty on long-term outcome has not been well described in the literature. We previously reported that while the long-term disease-free survival of patients over 80 years was similar to that of younger age groups, more than half of the older patients died of reasons unrelated to cancer [25]. In the present study, we aimed to investigate whether frailty severity classified by the 5-mFI score predicts the short- and long-term outcomes of elderly patients undergoing curative CRC surgery, including determining the cause of mortality and risk factors.

## Methods

We recruited 377 patients over 60 years of age who underwent elective colorectal resection with curative intent between January 2013 and December 2017 at Saint Mary’s Hospital. Seventy-eight patients were excluded because of stage IV CRC (*n* = 57) and emergency surgery (*n* = 21). In total, 299 patients over 60 years of age were retrospectively selected for our analysis: 248 patients with primary colon cancer and 51 patients with rectal cancer. All data were collected retrospectively. This study was approved by the Hospital Ethics Committee (approval number: 22–0612). Informed consent was obtained from all patients.

Patients were divided into three groups by the 5-mFI score. Group 1 (5-mFI = 0 or 1) had no frailty; group 2 (5-mFI = 2) was moderately frail; and group 3 (5-mFI ≥ 3) was severely frail. The 5-mFI score was determined by four comorbid conditions and one functional variable [16, 18], as follows: history of chronic obstructive pulmonary disease, congestive heart failure within 30 days postoperation, diabetes mellitus requiring oral agents or insulin, and hypertension requiring medication, as well as preoperative functional health status (independent, partially, or totally dependent). Each variable represents 1 point, for a total possible score of 5 points. A score of 2 or greater indicates frailty [16, 17]. The following data were collected from all patients:

a Patient characteristics: age, sex, preoperative body mass index (BMI), American Society of Anesthesiologists Physical Status score, comorbidities (cardiovascular disease, hypertension, cerebral disease, renal disease, respiratory disease, diabetes mellitus, liver disease, cancer in other organs), and blood test results (hemoglobin, albumin, carcinoembryonic antigen [CEA], and prognostic nutrition index [PNI] calculated as 10 × serum albumin [g/dl] + 0.005 × total lymphocyte count [/mm^3^] [26])
b Operation data: method (open or laparoscopic), amount of blood loss, duration of operation
c Tumor-related data: tumor location (right-sided/cecum to splenic flexure, left-sided/splenic flexure to sigmoid colon, or rectum), tumor size, tumor margin status (R0/R1), tumor differentiation (well, moderate/poor, mucinous), and tumor stage according to the 7th edition of the Union of International Cancer Control (UICC) [27]
d Postoperative outcomes:
  I. Short-term: postoperative hospital stay, morbidity defined as Clavien–Dindo classification ≥ III [28], mortality within 30 days, mortality within 1 year, and use of adjuvant chemotherapy
  II. Long-term: mortality at 5 years in addition to cause of death, from which overall survival and disease-free survival rates were determined. Cause of death was categorized as CRC-related or non-CRC-related, which comprised respiratory disease, cardiovascular disease, other primary cancer, and sepsis.

### Statistics

Values are expressed as frequencies and percentages. Differences in the categorical variables among the three frailty groups were analyzed using the chi-square test or Fisher’s exact test as appropriate. The duration of hospital stay was expressed as mean ± standard deviation and was analyzed using the Kruskal–Wallis test. Overall and disease-free survival rates were analyzed with the Kaplan–Meier method using the log-rank test. Overall survival was defined as the period from the date of surgery to the date of last follow-up or death. Disease-free survival was defined as the duration from surgery to the date of CRC recurrence. Univariate analysis was performed using the Cox proportional hazards model with survival time analysis as the dependent variable. Statistically significant variables were subsequently tested by multivariate analysis to determine the association between the individual determinants and death from CRC- and non-CRC-related causes, and the effect of each variable was assessed by the hazard ratio and 95% confidence interval. A significant *p*-value was set at *α* < 0.05. In the log-rank test, when comparing the three groups, all paired comparisons were adjusted for the *p*-value in multiple testing using the Holm method [29]. All statistical analyses were performed using the JMP software package (version 13.0; SAS Institute Inc., Cary, NC, USA).

## Results

### Preoperative characteristics of the colorectal cancer patients

Differences in preoperative features between the three frailty groups by the 5-mFI score are shown in Table 1. Of the 299 patients, 164 patients (54.9%) comprised group 1, 91 patients (30.4%) comprised group 2, and 44 patients (14.7%) comprised group 3. Regarding the 5-mFI variables, the frequency of a history of chronic heart failure, hypertension, diabetes, and dependence in activities of daily living increased in direct proportion to the 5-mFI score, while there was no significant difference for a history of chronic obstructive pulmonary disease between the three groups. High 5-mFI score was significantly associated with older age, high frequency of comorbidities, and high American Society of Anesthesiologists Physical Status score. There was no significant difference in sex, BMI, or dysphagia before surgery, and laboratory studies revealed no significant difference in PNI, hemoglobin, albumin, or CEA.

**Table 1:** Preoperative characteristics of CRC patients classified by 5-mFI score

		Group 1	Group 2	Group 3	
Characteristic	*n*	5-mFl: 0, 1 (*n* = 164), *n* (%)	5-mFI: 2 (*n* = 91), *n* (%)	5-mFI: ≥ 3 (*n* = 44), *n* (%)	*p*-value
Age (years)					
60–79	218	128 (78.0%)	65 (71.4%)	25 (56.8%)	0.022*
≥ 80	81	36 (22.0%)	26 (28.6%)	19 (43.2%)	
Sex					
Male	172	88 (54.3%)	57 (62.6%)	27 (61.4%)	0.325
Female	127	76 (45.7%)	34 (37.4%)	17 (38.6%)	
5-mFI variables					
History of CHF	64	13 (7.9%)	26 (28.6%)	25 (56.8%)	< 0.001*
Hypertension	149	53 (32.3%)	61 (67.0%)	35 (79.5%)	< 0.001*
History of COPD	20	9 (5.5%)	7 (7.7%)	4 (9.1%)	0.634
Diabetes	90	17 (10.4%)	43 (47.3%)	30 (68.2%)	< 0.001*
Partial or total dependence	79	19 (11.6%)	33 (36.3%)	27 (61.4%)	< 0.001*
Comorbidities					< 0.001*
None	56	55 (33.5%)	0 (0%)	1 (2.3%)	
One or more	243	109 (66.5%)	91 (100%)	43 (97.7%)	
ASA-PS class					< 0.001*
1	83	80 (48.8%)	2 (2.2%)	1 (2.3%)	
≥ 2	216	84 (51.2%)	89 (97.8%)	43 (97.7%)	
BMI (kg/m^2^)					0.166
< 25	233	133 (81.1%)	70 (76.9%)	30 (68.2%)	
≥ 25	66	31 (18.9%)	21 (23.1%)	14 (31.8%)	
PNI					0.340
< 40	85	41 (25%)	29 (31.9%)	15 (34.1%)	
≥ 40	214	123 (75%)	62 (68.1%)	29 (65.9%)	
Hemoglobin (g/dl)					0.367
< 11	124	63 (38.4%)	39 (42.9%)	22 (50.0%)	
≥ 11	175	101 (61.6%)	52 (57.1%)	22 (50.0%)	
Albumin (g/dl)					0.351
< 3.5	98	48 (29.3%)	33 (36.3%)	17 (38.6%)	
≥ 3.5	201	116 (70.7%)	58 (63.7%)	27 (61.4%)	
CEA (ng/ml)					0.086
< 5	173	96 (58.5%)	57 (62.6%)	20 (45.5%)	
≥ 5	126	68 (41.5%)	34 (37.4%)	24 (54.5%)	
Dysphagia before surgery					0.8603
Positive	4	2 (1.2%)	1 (1.1%)	1 (2.3%)	
Negative	295	162 (98.8%)	90 (98.9%)	43 (97.7%)	

*CRC* colorectal cancer, *5-mFI* five-item modified frailty index, *CHF* congestive heart failure, *COPD* chronic obstructive pulmonary disease, *ASA-PS* American Society of Anesthesiologists Physical Status, *BMI* body mass index, *PNI* prognostic nutrition index, *CEA* carcinoembryonic antigen

^*^Statistically significant

Table 2 shows the CRC surgical data and tumor characteristics by the 5-mFI category. There were no significant differences in the amount of blood loss or the duration of operation; however, laparoscopic surgery was performed less often in patients as the 5-mFI score increased. Investigation of tumor-related factors revealed that while UICC tumor stage was higher in patients with a higher 5-mFI score compared with those with a lower score, there was no significant difference in tumor location, tumor size, tumor differentiation, or tumor margin status between the groups. Although all patients underwent surgery with curative intent, 24 specimens revealed residual tumor cells on the tumor margin (R1) across all groups: 10 (6.1%), 10 (11.0%), and 4 (9.1%) for groups 1, 2, and 3, respectively; R0 resection rates were 93.9%, 89.0%, and 90.9%, respectively. There was no significant difference in R0 resection rates between the three groups.

**Table 2:** Surgical data and tumor characteristics of patients with CRC classified by 5-mFI score

		Group 1	Group 2	Group 3	
Characteristic	*n*	5-mFI: 0, 1 (*n* = 164), *n* (%)	5-mFI: 2 (*n* = 91), *n* (%)	5-mFI: ≥ 3 (*n* = 44), *n* (%)	*p-*value
Surgical procedure					0.028*
Laparoscopic	237	136 (82.9%)	73 (80.2%)	28 (63.6%)	
Open	62	28 (17.1%)	18 (19.8%)	16 (36.4%)	
Blood loss (ml)					0.426
< 150	207	114 (69.5%)	66 (72.5%)	27 (61.4%)	
≥ 150	92	50 (30.5%)	25 (27.5%)	17 (38.6%)	
Operation time (min)					0.524
< 270	130	66 (40.2%)	43 (47.3%)	21 (47.7%)	
≥ 270	169	98 (59.8%)	48 (52.7%)	23 (52.3%)	
Tumor location					0.316
Right side (C–T)	126	64 (39.0%)	42 (46.2%)	20 (45.5%)	
Left side (D–Rs)	122	66 (40.2%)	36 (39.6%)	20 (45.5%)	
Rectum	51	34 (20.8%)	13 (14.2%)	4 (9.0%)	
Tumor size (mm)					0.486
≤ 40	169	90 (54.9%)	56 (61.5%)	23 (52.3%)	
> 40	130	74 (45.1%)	35 (38.5%)	21 (47.7%)	
UICC stage					0.038*
I/II	191	115 (70.1%)	53 (58.2%)	23 (52.3%)	
III	108	49 (29.9%)	38 (41.8%)	21 (47.7%)	
Tumor differentiation					0.392
Well and moderate	284	153 (93.3%)	88 (96.7%)	43 (97.7%)	
Poor and mucin	15	11 (6.7%)	3 (3.3%)	1 (2.3%)	
Tumor margin status					0.379
R0	275	154 (93.9%)	81 (89.0%)	40 (90.9%)	
R1	24	10 (6.1%)	10 (11.0%)	4 (9.1%)	

*5-mFI* 5-item modified frailty index, *UICC* Union of International Cancer Control

^*^Statistically significant

### Short-term outcomes

The duration of hospital stay after surgery (mean ± standard deviation) was 19.3 ± 14.5 days in group 1, 22.6 ± 18.1 days in group 2, and 24.9 ± 24.9 days in group 3, revealing that patients in group 3 required significantly longer hospital stays compared with patients in group 1 (*p* = 0.047). Table 3 shows the differences in the immediate postoperative course by 5-mFI score. Immediate postoperative morbidity rates for Clavien–Dindo classification ≥ III in groups 1, 2, and 3 were 9.1%, 14.3%, and 9.1%, respectively. There was no significant difference in immediate postoperative morbidity between the three groups; however, 30-day mortality (4.5%) in group 3 was significantly higher. Two patients in group 3 died within 30 days after surgery from heart failure and aspiration pneumonia, respectively. Adjuvant chemotherapy was administered postoperatively to all patients who were eligible for treatment: 68 (41.5%), 22 (24.2%), and 8 (18.2%); groups 1, 2, and 3, respectively.

**Table 3:**
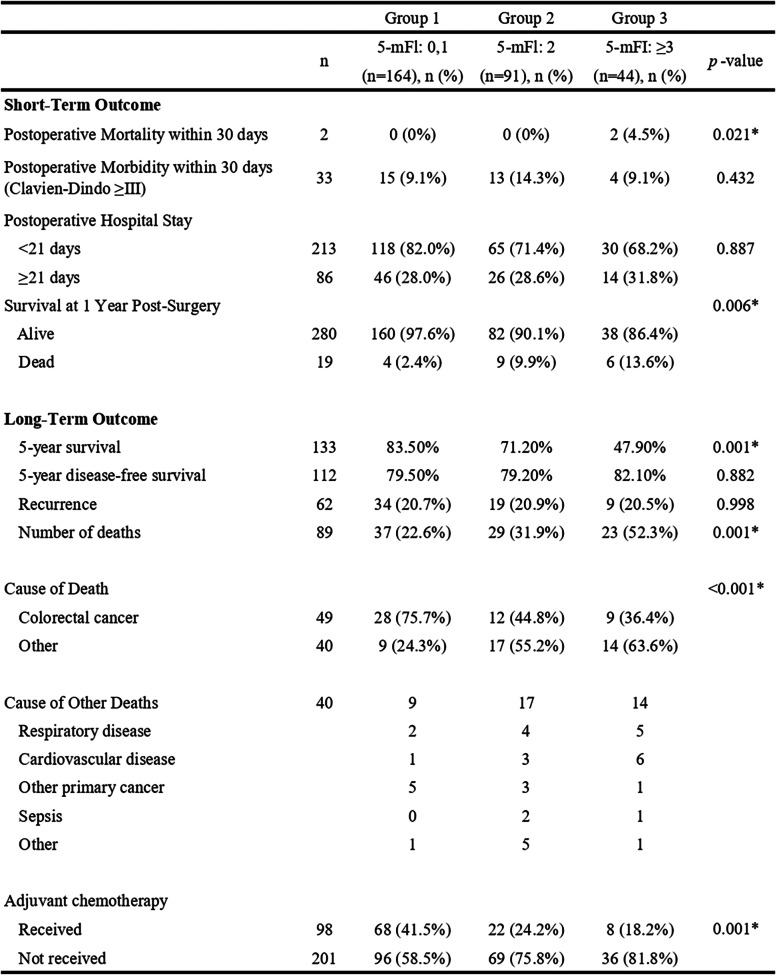
Short- and long-term outcome and cause of death in CRC patients classified by 5-mFI score

*CRC* colorectal cancer, *5-mFI* five-item modified frailty index

^*^Statistically significant

### Long-term outcomes

**Fig. 1:**
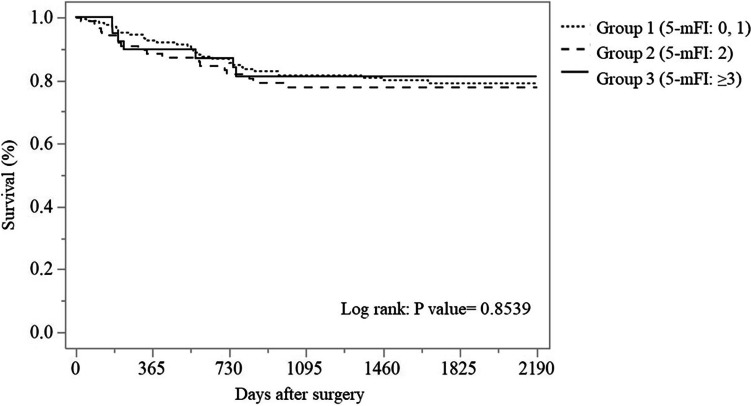
Disease-free survival in patients who underwent colorectal cancer surgery classified by the 5-mFI. 5-mFI, five-item modified frailty index

**Fig. 2:**
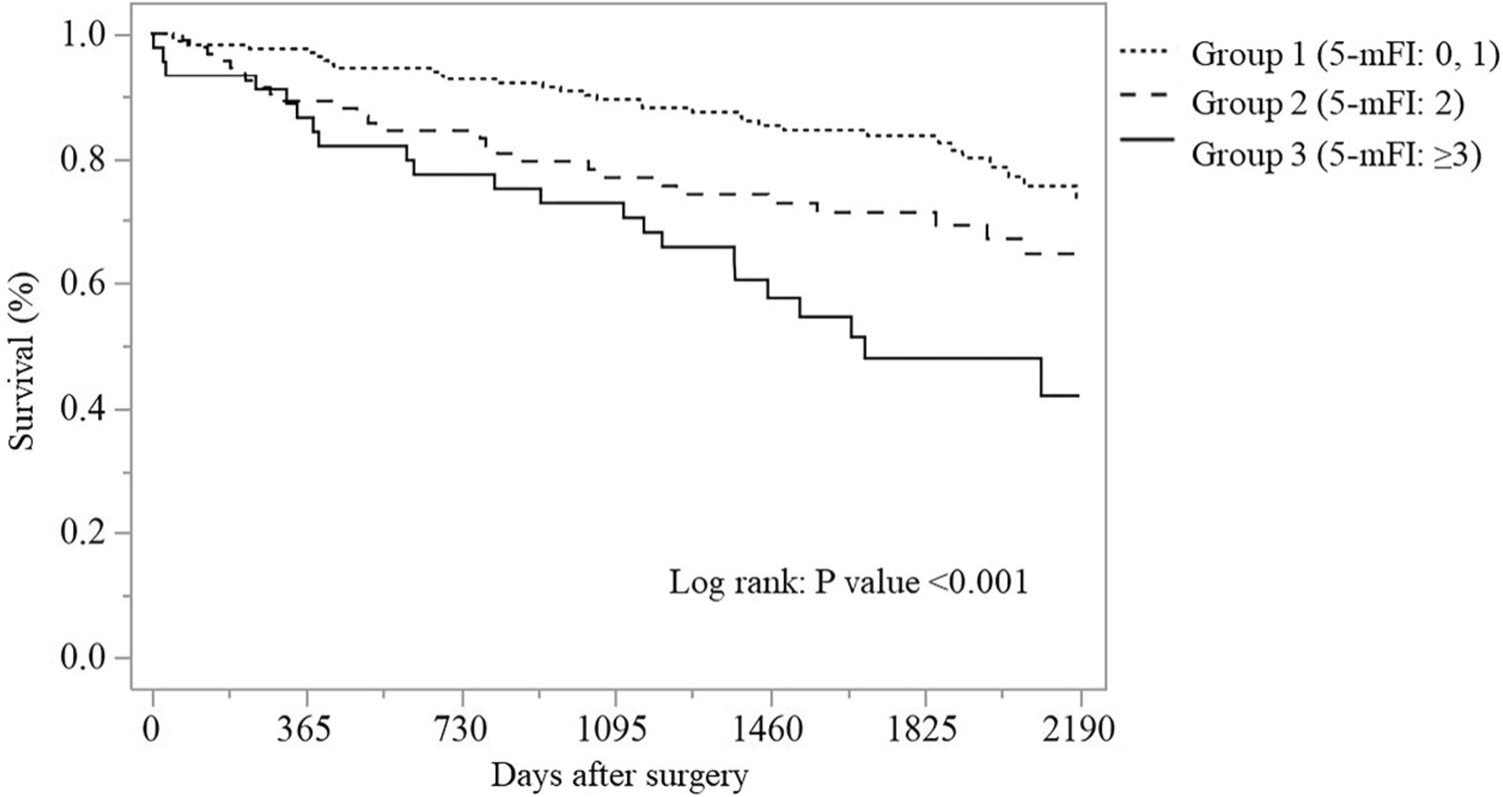
Overall survival in patients who underwent colorectal cancer surgery classified by the 5-mFI. 5-mFI, five-item modified frailty index

The median follow-up period for all groups was 57.3 months with a range of 0.2–114.8 months; 61.0 months in group 1, 54.8 months in group 2, and 47.4 months in group 3. During the follow-up, 62 patients (20.7%) developed local or distant metastases: 20.7% in group 1, 20.9% in group 2, and 20.5% in group 3, indicating no significant difference in disease-free survival between the three groups (Fig. 1). Of the 299 patients, 89 patients died: 37/164 (22.6%) in group 1, 29/91 (31.9%) in group 2, and 23/44 (52.3%) in group 3. A significantly higher mortality rate was observed in group 3 compared with groups 1 and 2. When classifying causes of death, 49/89 (55.1%) were from CRC and 40/89 (44.9%) were from other causes; 75.7% vs 24.3% in group 1, 44.8% vs 55.2% in group 2, and 36.4% vs 63.6% in group 3, respectively. A notable finding was that while 75.7% of non-frail patients (28/37; group 1) died from CRC, the majority of frail patients (*n* = 31/52 in group 2/3; approximately 60%) died from causes unrelated to CRC. The two primary noncancer causes of death were respiratory failure (29.0%) and cardiovascular disease (29.0%); other causes comprised primary cancer (12.9%), sepsis (7.1%), and others (7.1%). Although there was no significant difference between the three groups for disease-free survival, overall survival was significantly lower in group 3 (*p* < 0.001) (Table 3, Figs. 1 and 2). The survival rate in group 3 was lower than those in group 2 (*p* = 0.021) and group 1 (*p* < 0.001) by multiple pairwise comparison; there was no significant difference in survival between groups 1 and 2 (*p* = 0.070). The 5-year survival rates in groups 1, 2, and 3 were 83.5%, 71.2%, and 47.9%, respectively, revealing that the 5-year survival rate in patients with frailty (groups 2 and 3: 63.4%) was significantly lower than that in patients without frailty (group 1: 83.5%). The overall survival rates in groups 1, 2, and 3 were 61.3%, 32.3%, and 31.4%, respectively. There was one outlier in group 2 (survival: 3236 days, died of CRC). Then, we analyzed overall survival except the outlier, resulting in overall survival rates in groups 1, 2, and 3 of 61.3%, 64.1%, and 31.4%, respectively.

### Factors influencing cause of death

A univariate analysis of CRC-related deaths revealed that age, PNI, hemoglobin, CEA, tumor margin status, UICC stage, tumor differentiation, postoperative hospital stay, morbidity, and operative blood loss were significant factors influencing the cause of death. Multivariate analysis showed that UICC stage, CEA, tumor differentiation, and margin status were important risk factors for death (Table 4). A univariate analysis of non-CRC-related death revealed that age, 5-mFI score, PNI, surgical procedure, tumor location, postoperative hospital stay, morbidity, and operation time were significant risk factors. Age, 5-mFI score, and postoperative hospital stay were significant risk factors for non-CRC-related death in the multivariate analysis.

**Table 4:**
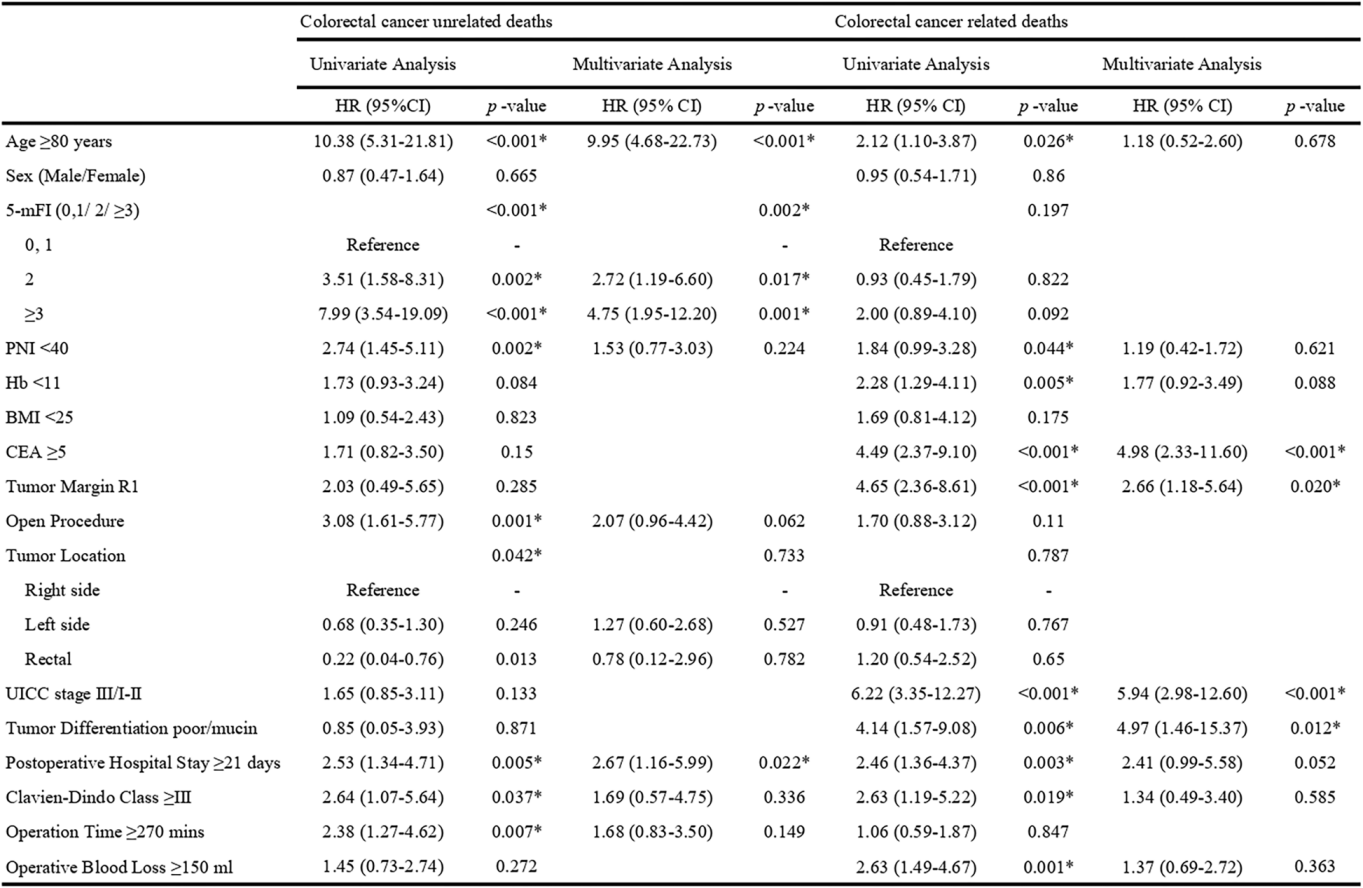
Univariate and multivariate analyses of survival after colorectal cancer surgery

*HR* hazard ratio, *CI* confidence interval, *5-mFI* five-item modified frailty index, *PNI* prognostic nutrition index, *Hb* hemoglobin, *CEA* carcinoembryonic antigen, *UICC* Union of International Cancer Control

^*^Statistically significant

## Discussion

In the present study, we aimed to determine the effect of frailty on the short- and long-term survival of elderly patients after CRC surgery. The 5-mFI score was significantly associated with 30-day mortality, 1-year mortality, and longer hospital stay, although there was no significant difference in 30-day morbidity between the three groups. Regarding long-term outcomes, higher 5-mFI score was associated with lower 5-year survival compared with a lower 5-mFI score. While most non-frail patients died of CRC, more than half of the deaths in frail patients were unrelated to CRC (primarily respiratory failure and cardiovascular disease). Advanced tumor stage, elevated CEA, undifferentiated tumor, and R1 resection were identified as risk factors for CRC-related death, while advanced age, high 5-mFI score, and long postoperative hospital stay were important risk factors for non-CRC-related deaths.

Frailty is considered a state of increased vulnerability resulting from a decline in physiological function across multiple organs [1]. Frailty has increasingly been recognized as a high-risk state predictive of adverse outcomes. However, frailty is considered to be separate from aging and comorbidity [30], and much research on frailty has been performed worldwide in the effort to improve comprehensive geriatric assessments in older adults with cancer [15, 31, 32]. Various frailty predictive models have been developed since 2001; however, early models are cumbersome and difficult to use [9, 17]. In 2013, the simpler 11-mFI, which can be used easily during history-taking and physical examination, was proven to be correlated with mortality and morbidity [9] and is currently widely used clinically [12–14]. The even simpler and less time-consuming 5-mFI was developed recently, and there is robust research comparing the 5-mFI and 11-mFI for predicting postoperative outcomes [15–17]. A weighted Kappa statistic showed strong agreement between the 5-mFI and 11-mFI in these studies [16, 17]. Dauch et al. reported that the 5-mFI was 88% comparable in predicting frailty compared with the 11-mFI [33]. Therefore, research has proven that the 5-mFI and 11-mF have similar predictive abilities. Furthermore, gathering data for the 5-mFI and using the index in practice is relatively easy, lending itself to clinical use in the geriatric population [15]. With these considerations, we chose the 5-mFI as the assessment tool for this study.

The trend in current studies on frailty and postoperative outcomes is to compare data between two groups: patients with and without frailty [3, 34, 35] or three groups: nonfrail, prefrail, and frail [17, 18, 31]. Dasgupta et al. used the Edmonton Frail Scale, with scores ranging from 0 (not frail) to 17 (very frail), in patients ≥ 70 years of age who underwent noncardiac surgery [36]. The study showed that patients with scores > 7 had increased complications compared with those with scores < 4 [36]. Mosquera et al. classified the 11-mFI into four groups, as follows: no frailty (0 points), mild frailty (1 point), moderate frailty (2 points), and severe frailty (≥ 3 points) in thoracoabdominal operations, and found that increased frailty was strongly associated with increased mortality and major complications [14]. We classified patients into three groups on the basis of the 5-mFI score, as follows: group 1, nonfrail (5-mFI = 0 or 1), group 2: moderately frail (5-mFI = 2), and group 3: severely frail (5-mFI ≥ 3) to investigate postoperative outcomes. This three-group categorization was expected to help refine our judgment when choosing to proceed with curative CRC surgery in patients with severe frailty.

Our study showed that patients with severe frailty had significantly poorer short-term postoperative outcomes, such as 30-day/1-year mortality and longer hospital stays compared with nonfrail patients and those with moderate frailty. These findings were similar to those in previous studies [4, 17, 37], although our study showed no significant difference in 30-day morbidity. Other research has demonstrated that frailty predicts poor postoperative outcomes, including a higher risk of postoperative morbidity [4, 13, 17, 18, 37], higher 30-day mortality [4, 13, 17, 37, 38], longer hospital stay [4, 13, 17, 37], and higher rates for readmission [4, 13, 17, 37], discharge to a facility other than home [17], and reoperation [13, 17]. Numerous studies using the 5-mFI also indicated that the 5-mFI score was associated with poor short-term postoperative outcomes, such as 30-day mortality and 30-day morbidity [17, 39]. Notably, these studies reported short-term outcomes but lacked long-term data and cause of death. In comparison, our study showed that the 5-mFI score was significantly associated with not only poor short-term outcomes, but also poor long-term outcomes and risk factors for mortality unrelated to CRC. Additionally, we found that long-term survival was negatively associated with the 5-mFI score.

Curative resection and adjuvant treatment are the standard treatments for CRC; however, there has been debate regarding whether surgery with curative intent should be performed for elderly patients. Historically, elderly patients with CRC have been hesitant to undergo curative surgery owing to the associated high morbidity and mortality and have received less aggressive treatment or even undertreatment [40–42]. Recently, owing to improved perioperative management and the use of laparoscopy, the 30-day survival rate for CRC surgery patients aged ≥ 65 years has decreased from 6 times as high to 3 times as high as that of younger age groups [25]. However, long-term survival in older patients has not been well described or analyzed. In the present study, contrary to the decrease in overall survival, the 5-year disease-free survival rate was similar between the three groups, indicating oncological benefit to patients irrespective of the degree of frailty. Our study showed that risk factors for CRC-related death were R1 resection, advanced tumor stage, CEA ≥ 5 ng/mL, undifferentiated tumor status, and longer postoperative hospital stay, which were findings similar to those in previous reports [43–45].

Studies on the effect of frailty on long-term survival in elderly patients after CRC surgery are limited and controversial. Artiles–Armas et al. reported that frailty did not affect 5-year survival [34], while Ommundsen et al. found that 5-year survival in frail patients (24%) was significantly lower than that in nonfrail patients (66%) [35]. Our study indicated that 5-year survival in frail patients (groups 2 and 3: 63.4%) was significantly lower than that in nonfrail patients (group 1: 83.5%). Artiles–Armas et al. and Ommundsen et al. studied patients aged ≥ 70 years with tumors ranging from stage I–IV [34, 35]; stage IV tumors accounted for 4.7% and 11.8% of the patients, respectively [34, 35]. In our study, we included patients aged ≥ 60 years with stage I–III tumors and excluded emergency patients and those with stage IV tumors. The frequency of the laparoscopic approach was 79.3% in our study and 29.2–38.9% in the mentioned previous studies [34, 35]. Compared with the survival in our study, the lower survival in the previous studies might have resulted from the lower rate of laparoscopy as well as the inclusion of stage IV tumors. Neither previous study reported the cause of death in detail. Moreover, the high frequency of the laparoscopic approach in our study may be related to the lack of a significant difference in short-term morbidity between the frailty groups [43].

In the present study, we found that despite similar disease-free survival between the frailty groups, long-term survival was markedly lower in more severely frail patients compared with those with moderate frailty. Additionally, more than half of the deaths in more severely frail patients were unrelated to CRC; deaths were from other causes, such as respiratory failure and cardiovascular disease. This was the reason why long-term survival was lower in more severe frail patients compared with less severe frail patients, despite the lack of a significant difference in DFS between the groups. KRAS and BRAF mutations are significantly associated with both DFS and overall survival [46]. We will investigate survival including KRAS and BRAF status in elderly patients with CRC surgery in future studies. Advanced age, 5-mFI score, and postoperative hospital stay were independent risk factors for non-CRC-related mortality from respiratory failure and cardiovascular disease in a previous study [25].

The 5-mFI score has been reported to be a preoperative predictor of postoperative outcomes after surgery for liver resection, trauma, nephrectomy, breast reconstruction, spinal disorders, bladder cancer, and complex head and neck disorders [19–23, 33, 47]. In the present study, high 5-mFI score was identified as an important risk factor for non-cancer-related deaths after CRC surgery. Preoperative assessment using the 5-mFI score may help reliably predict both non-CRC-related death as well as postoperative complications, and preoperative identification of frailty may allow for improved decision-making when selecting elderly patients for CRC surgery. The 5-mFI score is easy to calculate and use as a standard assessment for frailty in patients who may undergo surgery. Multimodal prehabilitation comprising exercise, nutrition, and counseling may improve frailty and help patients avoid postoperative complications and mortality [48, 49].

The present study has several limitations. First, this was a retrospective study that lacked assessment of postoperative quality of life. Second, patients with stage IV CRC and those who underwent emergency surgery were excluded, which further reduced the relatively small sample size. Third, advanced tumor stage was disproportionately represented in patients in group 3, which was the severe frailty group. The strengths of this study are the availability of detailed perioperative information on the population that underwent CRC surgery, as well as the promising long-term outcomes that were identified. Additionally, this study highlighted the potential for preoperative frailty evaluation using the 5-mFI to become a powerful tool in determining which elderly patients might receive the most benefit from surgery.

## Conclusions

The 5-mFI score can be a useful predictor of certain short-term outcomes (30-day and 1-year mortality), long-term outcomes (5-year survival), and mortality unrelated to CRC. Additionally, long-term survival was shown to be negatively associated with the 5-mFI score. Appropriate preoperative assessment using the 5-mFI can be a potential tool not only for the selection of elderly patients for CRC surgery but also for the identification of patients who may benefit from a prehabilitation program before surgery.

## Data Availability

The datasets generated or analyzed during the current study are not publicly available because of the data protection of the database but are available from the corresponding author on reasonable request.

## Abbreviations

5-mFI: Five-item modified frailty index
CRC: Colorectal cancer
UICC: Union of International Cancer Control
BMI: Body mass index
CEA: Carcinoembryonic antigen
PNI: Prognostic nutrition index
